# Supplementary material for: Targeted Radionuclide Therapy Using Auger Electron Emitters: The Quest for the Right Vector and the Right Radionuclide
Source: Pharmaceutics. 2021 Jun 29;13(7):980. doi: 10.3390/pharmaceutics13070980 (PMC8309076; doi:10.3390/pharmaceutics13070980)
Supplement: Supplementary file 1 [file pharmaceutics-13-00980-s001.zip › pharmaceutics-1213236-supplementary.pdf]

pharmaceutics

Supplementary Materials: Targeted Radionuclide Therapy Using Auger Electron Emitters: The Quest for the Right Vector and the Right Radionuclide

Malick Bio Idrissou, Alexandre Pichard, Bryan Tee, Tibor Kibedi, Sophie Poty and Jean-Pierre Pouget

Supplementary figure

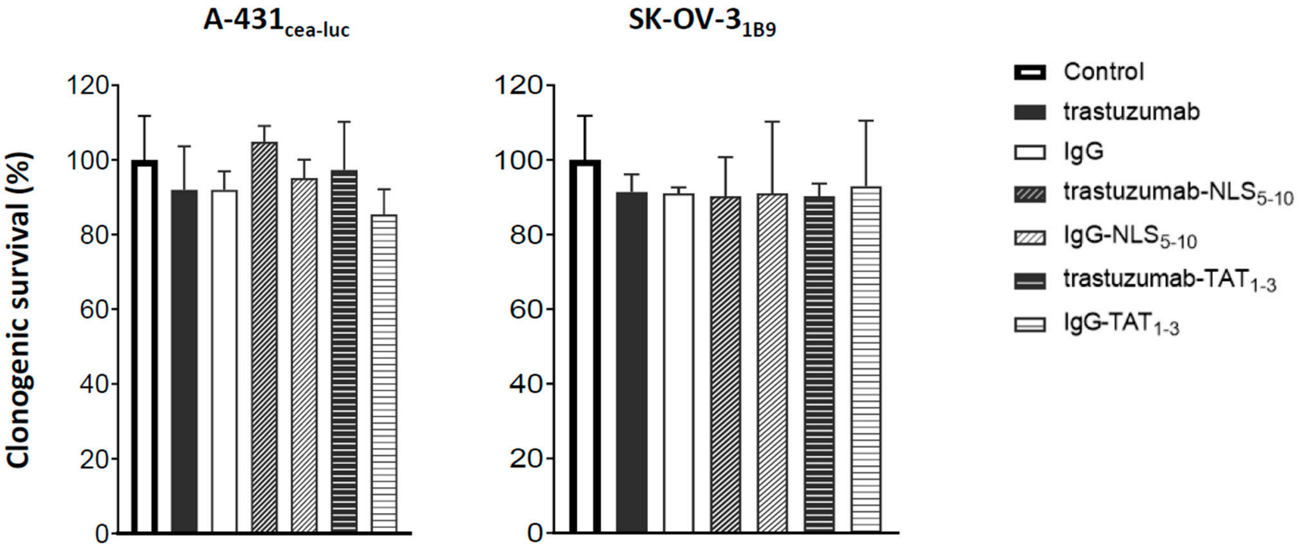

Figure S1. In vitro clonogenic cell death of unlabelled immunoconjugates. Clonogenic survival was assessed in A-431$_{CEA-luc}$ and SK-OV-3$_{1B9}$ cells 12 days after a 48 h exposure to trastuzumab, In-IgG, trastuzumab-NLS$_{5-10}$, IgG-NLS$_{5-10}$, trastuzumab-TAT$_{1-3}$ or IgG-TAT$_{1-3}$ (corresponding to the mAb mass added in the ^{111}In-treated 4 MBq/mL groups). Data are the mean ± SD. Experiments were performed at least three times in triplicate.
